# Introducing the Neuroplastic Narrative: a non-pathologizing biological foundation for trauma-informed and adverse childhood experience aware approaches

**DOI:** 10.3389/fpsyt.2023.1103718

**Published:** 2023-05-22

**Authors:** Haley Peckham

**Affiliations:** ^1^Centre for Mental Health Nursing, School of Health Sciences, University of Melbourne, Carlton, VIC, Australia; ^2^Department of Psychology, University of Exeter, Exeter, United Kingdom

**Keywords:** trauma-informed, adverse childhood experiences, neuroplasticity, Life History Theory, Attachment Theory, personality disorder, complex trauma, social justice

## Abstract

Most people accessing mental health services have adverse childhood experiences (ACEs) and/or histories of complex trauma. In recognition of this, there are calls to move away from medical model approaches and move toward trauma-informed approaches which privilege the impact of life experience over underlying pathology in the etiology of emotional and psychological suffering. Trauma-informed approaches lack a biological narrative linking trauma and adversity to later suffering. In its absence, this suffering is diagnosed and treated as a mental illness. This study articulates the Neuroplastic Narrative, a neuroecological theory that fills this gap, conceptualizing emotional and psychological suffering as the cost of surviving and adapting to the impinging environments of trauma and adversity. The Neuroplastic Narrative privileges lived experience and recognizes that our experiences become embedded in our biology through evolved mechanisms that ultimately act to preserve survival in the service of reproduction. Neuroplasticity refers to the capacity of neural systems to adapt and change. Our many evolved neuroplastic mechanisms including epigenetics, neurogenesis, synaptic plasticity, and white matter plasticity allow us to learn from, and adapt to, past experiences. This learning and adaption in turn allows us to better anticipate and physiologically prepare for future experiences that (nature assumes) are likely to occur, based on past experiences. However, neuroplastic mechanisms cannot discriminate between experiences; they function to embed experience regardless of the quality of that experience, generating vicious or virtuous cycles of psychobiological anticipation, to help us survive or thrive in futures that resemble our privileged or traumatic pasts. The etiology of suffering that arises from this process is not a pathology (a healthy brain is a brain that can adapt to experience) but is the evolutionary cost of surviving traumatizing environments. Misidentifying this suffering as a pathology and responding with diagnosis and medication is not trauma-informed and may cause iatrogenic harm, in part through perpetuating stigma and exacerbating the shame which attends complex trauma and ACEs. As an alternative, this study introduces the Neuroplastic Narrative, which is situated within an evolutionary framework. The Neuroplastic Narrative complements both Life History and Attachment Theory and provides a non-pathologizing, biological foundation for trauma-informed and Adverse Childhood Experience aware approaches.

## 1. Introduction

Many theorists and clinicians assume that abuse and complex trauma affect the developing brain and associated physiological systems in ways that are harmful and lead to abnormalities and psychopathology. The medical model and psychiatry take pathology as a premise and respond with diagnoses and treatments for the (mental) illness. Operating from dramatically different paradigms, both evolutionary theorists and consumers of mental health services challenge this perspective. While there is no doubt that abuse and complex trauma have a biological effect (1–3), evolutionary theory, the unifying theory of biological systems, suggests that a pre-emptive assumption of harm and pathology obscures the identification of adaptive mechanisms and forecloses a more nuanced exploration of the effects of trauma and abuse and the causes of psychological suffering (4–6). Evolutionary theory reveals that suffering is not a necessary indicator of dysfunction, but may, counterintuitively, indicate a functioning adaptation (7). Attempts to label and treat distress and suffering as a pathology can cause iatrogenic harm. To avoid such iatrogenic harm, psychiatry and medicine may engage at a deeper level with evolutionary theory (8–11) and contemporary neuroscience which reveal a multitude of mechanisms that transduce lived experience into adaptive biological changes (12–17).

A similar call to action for psychiatry is echoed by the consumer recovery movement (18). Many consumers who have experienced complex trauma and have had their suffering pathologized and diagnosed are calling for mental health services to become trauma-informed. They call for an approach that de-emphasizes diagnosis and the narrative of pathology that accords with the prevailing medical model view in psychiatry. They call instead for an approach that emphasizes the attempts of survivors of complex trauma to adapt to and survive in early traumatizing environments (19–21). Recognizing that our earliest experiences shape our brains and calibrate our physiology to maximize our chances of being able to survive and reproduce in our anticipated futures validates the biological impact of experiences of adversity and complex trauma. This is axiomatic for a trauma-informed approach and the Neuroplastic Narrative supports this emphasis.

In this study, the reader is invited to engage with a conceptual digression that draws on an ecological rather than pathological framing. The Neuroplastic Narrative is not intended to replace the medical model but to sit as an alternative framework from which to understand and respond to emotional and psychological suffering, the common goal of both approaches. The reality of mental illness where there is an identifiable pathology is not denied, but where the pathology is yet to be identified or the diagnosis is contested, the Neuroplastic Narrative may offer both the people seeking help and the people seeking to help them an alternative perspective.

## 2. Evolutionary perspectives

### 2.1. Adaptation occurs over many lifetimes and within a single lifetime

In contrast to traditional views that segregated the effects of nature (genes) and nurture (the environment), a contemporary evolutionarily informed view recognizes that there is an intricate interplay between the nature (genotype) of the organism and nurture (environment) that produces its phenotype (observable traits). This is succinctly captured by Martin Teicher, a researcher, on the effects of child abuse and maltreatment, “*Brain development is directed by genes but sculpted by experiences”* (p. 652) (2). Genes provide us with evolution's best guess of what we will need to survive in any environment (such as the capacity to learn a language), and experience hones those capacities into *precisely* what we need to survive in the environment we live in (to speak the language we most hear spoken). “*Genes… are the variable set of control elements that natural selection changes or tunes over evolutionary time so that, in developmental time, the resulting interaction of the organism's genes and its environmental regularities causes the development of biologically functional structure*” (p. 459) (4). In other words, we are stable enough in our environment because our genetic inheritance has been sculpted by natural selection *over many* lifetimes, but we are “plastic” enough to adapt to our environment *within* our lifetime.

Developmental plasticity allows individuals of the same genotype to mature into different phenotypes because they have been changed by environmental conditions encountered during development (22). Developmental plasticity is conserved across taxonomic groups indicating that, across populations, the individual organism's capacity to adapt to the particular environment they encountered during development enhances their ability to survive and reproduce in a similar environment (23, 24). Neuroplasticity refers to the capacity of neural systems to adapt and change in response to experience and injury (25). Neuroplasticity, like developmental plasticity, is greatest during development but neuroplasticity is a property of living neural systems, a dynamic process that continues throughout life into aging (26, 27). The genetic programs that support plasticity simultaneously confer both potential and vulnerability because the potential will be realized—made real, for better or worse, by our environment. This is so whether we strike it enriched and supportive or impoverished and hostile (28).

The crucial role of experience in structuring the developing brain and nervous system was first identified by Hubel and Wiesel (29) Nobel Prize winners, in their studies on the visual system. Their experiments revealed that there was a “critical period” in the development of the visual system which was dependent on the experience of being able to see (29). The profound need for experience to build neural pathways and myelinate tracts of children's brains can also be inferred from the devastating effects of the privation of experience through institutional neglect (30). The power of experience to protect against pathology has also been demonstrated by enriched environment protocols that delay the onset of Huntington's disease symptoms (31) and rescue memory deficits in mice (32). Bruce McEwen, neuroscientist and stress researcher writes: “*The brain is the central organ for adaptation to experiences*” (p. 56) (33) and a multitude of neuroplastic mechanisms that transduce experience in the environment into changes that shape the structure and function of the brain and nervous system have evolved to facilitate this. Experiences shape brains, and they are supposed to because a healthy brain is a brain that can adapt to experience (34).

### 2.2. Mechanisms of neuroplasticity

Adaptation to the experienced environment occurs via neuroplastic mechanisms including (but not limited to) epigenetic mechanisms at the level of the DNA that alter gene expression rates; neurogenesis, the birth of new neurons and their integration into the existing network; synaptic plasticity, which connects neurons creating neural pathways; and white matter plasticity, myelination of active neural tracts that span regions of the brain. These mechanisms evolved over evolutionary time to embed early experience under our skin in developmental time, improving the chances of surviving and reproducing in an ecological niche similar to the one from which the early experiences were drawn.

#### 2.2.1. Epigenetics

Epigenetics is the study of how changes in the external or internal environment impact gene expression. “Epi” is a Greek prefix meaning “above” or “over”, and epigenetics identifies mechanisms above or over genes that regulate their expression serving phenotypic plasticity. These enhance our adaptation to, our fitness within, the current and/or putative future environment (35). These epigenetic changes may persist in the organism for a short or long time and the epigenetic change itself may be inherited, changing gene expression within, and sometimes beyond, the next generation. For comprehensive reviews on epigenetic mechanisms related to complex trauma and adversity, see (36–38).

A powerful illustration of the epigenetic process is seen in rodent stress studies. The hypothalamic pituitary adrenal axis mediates the response to stressful stimuli by releasing corticotrophin-releasing hormone (CRH), adrenocorticotrophic hormone (ACTH), and cortisol (corticosterone in rodents) which acts through the mineralocorticoid and glucocorticoid receptors. In rodents, the sensitivity of the HPA axis is calibrated by early experience via epigenetic mechanisms (39). Rat pups that receive enhanced licking and grooming by their mothers in the first 10 days of life have a more highly expressed glucocorticoid receptor gene, a faster and more efficient negative regulation (switching off) of their stress response and consequently reduced responses to acute restraint stress in adult life (40). A subsequent landmark study revealed that it was an epigenetic mechanism that transduced the experience of maternal care into an enduringly sensitive and responsive HPA axis (41). High levels of maternal care behaviors (licking and grooming) increased the permissive epigenetic marking on the glucocorticoid receptor gene, leading to a greater number of glucocorticoid receptors being expressed that both swiftly mediate and efficiently terminate the effects of the circulating corticosterone (stress hormone) (41). Low levels of maternal care behaviors (less licking and grooming) lead to fewer glucocorticoid receptors being expressed which maintains and prolongs the stress response as the corticosterone is circulating for longer. The stress system has evolved to support our survival in stressful conditions, so both outcomes are adaptive if the quality of maternal care experienced accurately forecasts the challenge of the future environment. In a challenging and stressful environment, evolution's end of survival and reproduction may be served by a chronically activated, prolonged stress response that has been calibrated early in life by low levels of maternal care. This suggests that natural selection has attuned offspring to adapt to variations in parental care because parental care foreshadows future environmental conditions (41).

In humans, the epigenetic status of the glucocorticoid receptor gene is altered by experience as follows: *in utero* by intimate partner violence (42), by childhood abuse and maltreatment (43–45) in healthy adults with histories of trauma and abuse (46), and clinical populations with depression following early adversity (47) or a history of childhood emotional abuse (48). The epigenetic status of the glucocorticoid receptor gene is also altered in people diagnosed with borderline personality disorder (44, 49) and suicide completers, both with histories of complex trauma (43, 50).

#### 2.2.2. Neurogenesis

Neurogenesis is the process by which new neurons are generated and integrated into the existing neural network (51). In humans, the rate of adult neurogenesis in the hippocampus is sufficient for it to contribute to neuroplasticity, our capacity to learn and adapt to experience (52, 53) to forget what is no longer salient, integrate novel information into pre-existing contexts, and adapt affective behaviors (54). Across species, lived experience in the environment alters the rate and success of neurogenesis in adaptive ways. Both captive and wild food-caching black-capped chickadees have higher rates of neurogenesis in the autumn when they form memories of the locations where they have cached food, but the wild birds that are dependent on their memories of stored food generate new neurons at twice the rate of the captive birds (55). Black-capped chickadees in harsh environments, that are heavily reliant on cached food, have even higher rates of neurogenesis, suggesting the process of neurogenesis is responsive to the chickadees' experience of food scarcity in their local environment (56). In rodents, physical exercise and novelty tend to increase neurogenesis, and stressors (such as social defeat, inescapable foot shock, and restraint stress) reduce neurogenesis (57). Interestingly, mildly stressful experiences of novelty and complex enriched environments may enhance neurogenesis and be protective experiences against future stressful events acting as “stress inoculation” (58). Overall, environments providing rewarding and enriching opportunities seem to prime brains, through enhancing neurogenesis, to learn and explore. Conversely, experiences of restraint, social defeat, punishment, and impoverishment prime brains, through reducing neurogenesis, for safety and avoidance (57). Taken together, this suggests that what is salient about experiences that enhance neurogenesis, priming the brain for learning, is that there are unknowns that signify opportunities. In contrast, experiences that are known (such as being in an impoverished, unchanging environment or re-encountering a known threat or punishment) inhibit neurogenesis, perhaps because there is nothing new to be learned or explored.

A study on the effects of early life stress through maternal separation in rats further evidences that changes in gene expression and neurogenesis are meaningful and adaptive (59). Rat pups that had endured daily periods of separation from their mothers (3 h, days 2–14) performed better in a spatial learning task in adolescence (2 months) compared to the control group who had not been separated from their mothers. At the same time, as they exhibited this learning advantage, the rat pups experiencing maternal separation also had less epigenetic repression on their brain-derived neurotrophic factor (BDNF) gene (coding for a protein involved in learning and memory processes) and enhanced neurogenesis. However, when both groups of rats were tested on a new learning task at middle age (15 months), the situation reversed. At middle age the rats that been separated as pups performed less well on memory tasks, had more epigenetic repression on their BDNF gene, and reduced neurogenesis compared to the control group who had not undergone maternal separation. The authors concluded “*that early stress may transiently endow animals with a potential adaptive advantage in stressful environments but across a life span is associated with long-term deleterious effects”* (p. 2) (59). The experience of being without their mother had meaningfully changed the pups' neurobiology through epigenetics and neurogenesis, accelerating their capacity to learn their environment and consequently enhancing their chances of surviving through to reproductive age. The pups that did not have the experience of maternal separation did not need enhanced learning abilities because their mothers were present to support their survival in the environment. Rat pups appear to have evolved a biological contingency of accelerating cognitive development in response to variable early parental care. This serves the pups' survival, by adapting to the early environmental conditions experienced (41).

#### 2.2.3. Synaptic plasticity

Synaptic (or neural) plasticity and white matter plasticity are mechanisms of neuroplasticity that operate serially: experiences create and strengthen connections (synapses) between neurons forming neural pathways, and repeated experience leads to myelination of the most active neural pathways, which makes them more efficient and automatic (12). Synaptic plasticity is characterized by the quip “*cells that fire together wire together”* (p. 64) (60) and long-term potentiation, the strengthening of active synapses, has been accepted as the cellular basis for learning and memory since its discovery in 1972 (61). Many studies of synaptic plasticity were done in *Aplysia*, the Californian sea slug, by Nobel prize winner Eric Kandel and colleagues. The researchers used the experience of a benign or painful stimulus to moderate the reflexive gill withdrawal behavior of the sea slug and mapped the learned behavior change to the molecular level of the synapse, indicating that experience in the world alters structure and function at the level of the synapse (62). A more recent study in humans, looking at gray matter volume (synaptically dense unmyelinated areas), showed that London taxi drivers have larger posterior hippocampi (regions associated with spatial learning) than non-taxi drivers, consistent with the interpretation that their brains have become specialized for the task through real-world experience. The authors conclude, “*that there is a capacity for local plastic change in the structure of the healthy adult human brain in response to environmental demands”* (63). Gray matter volumes are also altered by childhood experiences of deprivation (64, 65) and maltreatment (66) including harsh corporal punishment (67), emotional (verbal) abuse (68), sexual abuse (69, 70), and witnessing domestic violence (71).

#### 2.2.4. White matter plasticity

White matter plasticity, activity-dependent myelination, or adaptive myelination all refer to a form of neuroplasticity that links experience in the world with changes to myelin or to the cells that do the work of myelination—oligodendrocytes (13, 72, 73). Myelination is the process of wrapping the axons of the neurons that make up neural pathways in myelin, which makes the transmission down that neural pathway faster and more efficient. Experience activates neural pathways, and the activation releases signaling molecules such as brain-derived neurotrophic factor which switches myelination into an activity-dependent mode (72, 74, 75). Repeated experiences repeatedly activate neural pathways driving activity-dependent myelination and make those neural pathways faster and more efficient, and the behavior or thought associated with that neural pathway more automatic. As white matter can be imaged, many studies demonstrate this in humans. Pianists have highly developed white matter in specific brain regions which correlates with the number of years that they have been playing (76). Learning to juggle alters white matter structure (77), as do reasoning and working memory training (78, 79), learning a second language (80), reading remediation (81), meditation, and mindfulness-based stress reduction (82, 83) and socioeconomic status (84, 85).

Social experience also impacts adaptive myelination. Studies in rodents indicate that myelination of the pre-frontal cortex is dependent on early social experience (86) and the effects of the experience of social isolation on adult pre-frontal cortex myelin can be reversed with subsequent experience of social re-introduction (87). In human adults, social network diversity (88) and secure attachment (89) are associated with white matter integrity in specific tracts. Conversely, children who are socially disadvantaged (84) or have experienced violence and social deprivation (90), institutional neglect (30), emotional (verbal) abuse (91), or witnessed domestic violence (92) also carry the signature of those experiences in their white matter. Positive socioemotional experiences in the form of high-quality foster care placements can mitigate the effects of prior experience of institutional neglect on the white matter tracts of children (64, 93).

Imaging studies that show experiences structurally shape brains, raise questions about psychiatric imaging studies that correlate structural brain changes with psychiatric diagnoses. In such studies, prior experiences, including those of childhood neglect and abuse, if not controlled for, may be an unrecognized confound, as the possibility that the structural changes are due to maltreatment rather than pathology cannot be discounted. Teicher observes that “*maltreatment has likely been an insidious confound in nearly all psychiatric imaging studies”* (p. 245) (3), which may produce ecophenotypic (environmentally caused) variants of psychiatric disorders that are clinically distinct (94) which could have significant policy and treatment implications.

### 2.3. Adverse childhood experiences

Our experiences shape our brains and physiology. Adaptation supports the survival of our species, so mechanisms have evolved, such as epigenetics, neurogenesis, synaptic plasticity, and white matter plasticity, that transduce our lived experience into adaptive biological changes. For so many mechanisms of neuroplasticity to have evolved indicates that the capacity to learn from and adapt to past experiences and to better anticipate the threats and opportunities we may have in the future enhances inclusive fitness, survival, and reproduction in our environment. But if this is true, how can we reconcile that with the evident suffering and decrease in life expectancy that comes from having had certain experiences or living in certain environments?

The landmark Adverse Childhood Experiences study undertaken in the late 90s by Kaiser Permanente and the Center for Disease Control in the USA. occurred in two waves involving 17,337 adults and correlated the number of categories of adverse childhood experiences (ACEs) with physical and mental health outcomes (95). In the 1998 published study, adverse childhood experiences (ACEs) were defined as sexual, physical, or psychological abuse, mother treated violently, family member ever imprisoned, substance abuse, or mental illness/suicidality in the home (96). Other studies include parental separation/divorce and physical or emotional neglect as categories of adversity (95). The study discovered a dose–response relationship between the number of categories of adversity experienced as a child and health risk factors for several of the leading causes of death. More than half of the respondents had encountered adversity, one in eight had more than four ACEs which at least doubled their chances of having sexually transmitted disease, heart attacks, cancer, stroke, or lung disease; tripled their chances of having more than 50 sexual partners over a lifetime; quadrupled their risk for depression; made them 5–10 times more likely to use drugs, inject drugs, or become an alcoholic; put them at 12 times the risk of dying by suicide (96); and more than six (of 8 possible ACE categories which included separation/divorce) reduced life expectancy by almost 20 years (97).

A systematic review and meta-analysis incorporating 37 international studies and 253,719 participants, published in *The Lancet* in 2017 echoed the findings of the ACE study with more than half of respondents having at least one ACE and just over one in eight having four or more ACEs. People with four or more ACEs were at two to three times the risk for cancer, heart disease, and lung disease; three to four times the risk for early sexual initiation, teen pregnancy, and having multiple sexual partners; and four to six times the risk for mental illness, sexually transmitted infections, and problematic alcohol use. They were 7–10 times more likely to misuse substances and violently harm themselves or someone else and 30 times more likely to attempt to end their lives (98). Taken together, these studies strongly associate early experiences of adversity and complex trauma with poor health and social outcomes, suggesting, but not demonstrating, causality. If the neuroplastic mechanisms introduced above increase the chances of survival and reproduction by helping individuals adapt to their environments and learn from past experiences, why do the experiences of adversity and trauma lead to suffering, an increase in physical and mental illness, and shortened life expectancy?

#### 2.3.1. Damage or adaptation?

Experiences shape brains and physiology but do stressful experiences such as adverse childhood experiences and complex trauma lead to pathology (diathesis-stress hypothesis) (99) or adaptation (plastic-adaptation hypothesis) (100)? Adaptation is defined here as “*biological machinery or process shaped by natural selection to help solve one or more problems faced by the organism”* (p. 3) (5). The selection pressures of stress including early life stress have been present throughout mammalian evolution. Out of these pressures, adaptive mechanisms would emerge via the process of natural selection, to safeguard survival and reproduction in stressful environments (2). The “plastic adaptation” hypothesis is that: the experience of early life stress forewarns of a stressful future and leads to biological changes that forearm us, safeguarding (as far as possible) survival and reproduction in a putative stressful future environment. “*Specifically we propose that childhood abuse alters the development of particular brain regions, in an experience-dependent plastic manner, to facilitate survival and reproduction in what seems, so far, to be a threatening and malevolent world”* (p. 653) (2). The suffering caused by adaptation may seem self-evidently pathological. However, in a dangerous and unpredictable environment, adaptations that support survival and reproduction offer a selective advantage, even at the cost of health and well-being (100–102). From this adaptationist perspective, suffering does not necessarily indicate pathology, because this perspective recognizes trade-offs between competing objectives, such as health and reproduction (5, 6, 103). As Dobzhansky observed “*nothing in biology makes sense except in the light of evolution”* (p. 86) (6), and Life History Theory, a contribution from evolutionary biology, offers a way to reconcile these counter-intuitive observations.

### 2.4. An evolutionary perspective: Life History Theory

Evolution is a non-sentient process of attrition of those less able to survive and reproduce that leaves those more able to survive and reproduce here to do so. Any heritable trait or mechanism that supports survival to reproductive age and reproduction is (by virtue of the process itself) going to become more prevalent in subsequent populations, even if it subsequently causes us to suffer and shortens our life expectancy. To be successful in evolutionary terms is to leave descendants ideally, many of them, all of which/whom would be highly competitive for available resources. However, organisms are limited both in what they can achieve and the resources they can draw on, and environments are highly variable so leaving descendents at all may be a challenge.

#### 2.4.1. Adaptation over evolutionary time

Life History Theory explains how solutions, in the form of strategies for survival and reproduction, emerge to solve the problem of how to best ensure the continuation of the genetic line given the pressures of a particular environment, and the limited resources available to, and constraints within, the organism (104). Environments and ecologies differ in a multitude of ways, but differences in harshness and unpredictability fundamentally impact the way organisms live and reproduce (105). To avoid extinction, organisms must leave descendants. In harsh unpredictable ecological niches, it is adaptive to produce the greatest number of descendants in the shortest possible time, thereby safeguarding against the possibilities of death or injury that leads to no descendants being left at all, an evolutionary dead end. This pressure is absent from environments that are characteristically safe and predictable. In a safe and predictable environment, where there is competition for the best resources, it is adaptive to produce highly competitive offspring. Strategies that either produce the greatest number of descendants in the least time or produce a smaller number of descendants that are highly competitive emerge from a range of traits and behaviors concerned with survival and reproduction. These traits and behaviors coherently group to enact the life history strategy that best fits the environment.

Survival-related traits and behaviors include birth size, growth rate, age and size at sexual maturity, number and size of offspring, birth spacing, parental investment in offspring, and lifespan (105, 106). These traits and behaviors vary according to how the organism allocates its resources. The organism, impacted by harshness and unpredictability in the environment allocates its limited resources to maximize the chance of leaving descendants. Fundamentally, in this ecological niche, should the organism prioritize growth now and reproduction later, or commence reproduction now in case it is not possible later? Should the organism maximize its mating effort and number of offspring, or have fewer offspring that require maximum parenting effort to ensure they are competitive (107)? Trade-offs must occur as options are mutually exclusive. These trade-off ‘decisions' drive resource allocation that leads to variation in coherently related traits and behaviors and a strategy for survival and reproduction emerges. The range of life history strategies for survival and reproduction fall along a continuum from fast to slow. These strategies have emerged from the process of natural selection to most likely be successful in environments arranged on a parallel continuum running respectively from high to low levels of harshness and unpredictability.

The variation in life history strategies across species is typically exemplified by the rabbit and the elephant. Rabbits are prey animals in their ecological niche and are beset by unpredictable, likely fatal events. They have low birth weight and rapid growth, are weaned by 6 weeks, and are ready to reproduce by 21 weeks. They mate indiscriminately and frequently, gestate for 1 month and give birth to up to 12 kits, and may become pregnant again within a week. Rabbits exemplify a fast life strategy, whereas elephants exemplify a slow life strategy. Elephants weigh approximately 100 kg at birth and take around 14 years to reach maturity. Gestation lasts for approximately 22 months and usually only one calf is born which suckles for 3 years and remains in very close proximity to its mother, family members, or allomothers, who invest in the care, protection, and socialization of the calf for around 8 years (106, 108).

Over evolutionary time, fast and slow strategies have emerged through the process of natural selection to be successful reproductive strategies in recurring environmental conditions of high and low harshness and unpredictability, respectively. This produces distinct life history strategies across species exemplified by the rabbit and elephant. However, variation in life history strategy occurs between species *and* within a species population (105). This suggests that the capacity that individuals have to adapt to their environment goes beyond Darwinian explanations of heritable variation (109). The extended capacity for systems to dynamically adapt to their local environment is described by complex adaptive systems theory (110). Complex systems adapt, learn, and continually improve *by “form*[ing] *and use*[ing] *internal models to anticipate the future* (p. 24) …*. balance*[ing] *exploration (acquisition of new information and capabilities) with exploitation (the efficient use of information and capabilities already available)”* (p. 26) (110). Adaptation occurs on two scales: over lifetimes and within them.

#### 2.4.2. Adaptation within a lifetime

**Figure 1:**
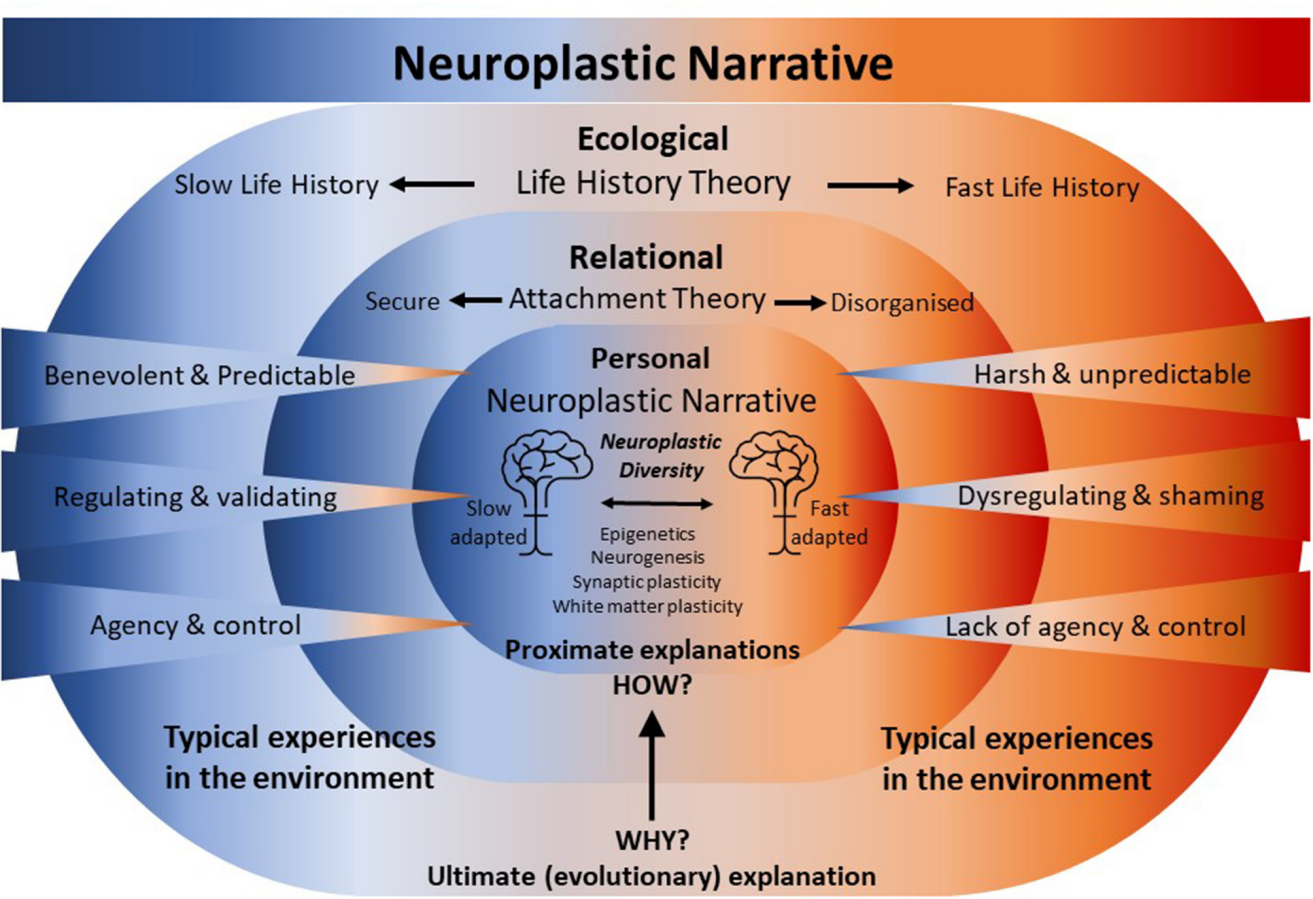
Neuroplastic narrative. Neuroplastic mechanisms act proximately to biologically embed experiences gained during development within a relational and ecological niche. This embedding of experience gradually specifies an attachment pattern and a life history strategy that ultimately calibrates the individual for survival and reproduction in the same or similar ecological niche. The attachment pattern and the life history strategy describe an orientation to, and calibration for, a future ecological and relational niche—an anticipation that is achieved through the embedding of past experiences by neuroplastic mechanisms. Experiences in the environment range over a “continuum of normal” between extremes. At one extreme, at the ecological level, experiences of harshness and unpredictability evoke fast life history traits and relational harshness and unpredictability mediated via dysregulating caregiving experiences and little sense of agency in a relational context may disorganize the formation of an habitual attachment pattern. At the other extreme, at the ecological level, experiences of a benevolent and predictable environment evoke slow life history traits and benevolent and predictable relational experiences mediated via regulating caregiving experiences coupled with a sense of agency in the relationship build a secure attachment. Experiences at the ecological and relational level are embedded “under the skin” shaping brain structure and function and calibrating physiology. As a consequence, brains and physiologies emerge that are faster or slow-adapted, generating Neuroecological Diversity.

Natural selection has favored mechanisms of plasticity that instantiate these qualities of complex adaptive systems. Mechanisms that support phenotypic plasticity allow individuals to tailor their life history strategy to the environmental conditions they encounter (111). Thus, life history strategies that emerge from complex systems are both heritable and influenced by the experience in the ecological niche encountered during development. “*The LH* [Life History] *strategies of individuals become adapted to their environments through two fundamental processes: evolution and development. Whereas natural and sexual selection adapt LH strategies to recurring environmental conditions encountered over evolutionary time, developmental experiences capture information that enables individuals to match LH strategies to environmental conditions encountered in their own lifetime”* (p. 254) (105). The developmental and neuroplastic mechanisms that *homo sapiens* have evolved enable adaptation to occur within an individual's lifetime, the capacity to adapt to the environment whilst living in it. These mechanisms invisibly shepherd development towards becoming a mature individual with traits and behaviors, especially survival and reproductive behaviors, that become the life history strategy most likely to lead to reproductive success in the ecological niche that has been encountered (see Figure 1).

In harsh and/or unpredictable environments where there is a significant risk of dying before reproducing, pre-natal and early indicative experiences of adversity drive developmental accommodations that make the best of a bad situation. The challenges of adversity, abuse, and neglect are physiologically and homeostatically dysregulating and predominate, which results in calibration to (and for) an environment that is dangerous and unpredictable. From an evolutionary perspective in a dangerous and unpredictable environment, it makes sense to accelerate development which drives precocious puberty, find a mate, and begin reproduction as early as possible (112–114). It also makes evolutionary sense to reproduce multiple times, increasing the number of offspring and therefore the chances that the genetic line will be continued rather than abruptly terminated by the same high-risk, dangerous, and unpredictable environment (101). Where future prospects are discounted in favor of an orientation *toward the self, in the present*, the development of particular characteristics and traits such as impulsiveness, risk-taking, antagonism, a tendency to exploit others and situations, sensation-seeking, and instant gratification also emerge, because by offering short-term gains, they serve survival within high-risk environments (115). The “*live fast die young”* fast life strategy is further characterized by a high birth rate, accelerated development, insecure or disorganized attachment, and younger age at first birth (101, 102, 113).

Contrasting with fast life conditions, in a safe and predictable environment where there is a low risk of dying before reproducing, fewer accommodations to adversity are made throughout development allowing a phenotype to unfold that has had fewer environmental impingements (116). The infant's early indicative experiences of being maintained at, or swiftly restored to, a comfortable homeostatic range predominate, which results in calibration to (and for) an environment that is safe and predictable. There is no rush to accelerate development to reproduce as the threats of injury and death are low. In this condition, at maturity, a long-term bond with a high-status mate can be sought and resources allocated to build a safe and predictable future, that is psychobiologically anticipated and can be planned for as the environment, has proven predictable. In a low-risk, safe, and predictable environment, there is a high chance that offspring will survive, so having fewer offspring and investing significantly in them ensures that they will be competitive (101). The capacity to reflect and consider, develop relational skills such as empathy, plan for the future, and defer rewards, all emerge from experiencing a safe predictable environment that calibrates us to, and for, a safe and predictable future (117). This is slow life history, characterized by slow growth, late maturation, secure attachment, reduced risk-taking, more acquired resources, fewer, larger offspring, slower aging, and a longer lifespan (100–102, 113).

### 2.5. The pathologization of the fast life strategist

Life History Theory predicts that individuals adapt to harsh, unpredictable environments by maximizing reproductive effort to ensure descendants are left despite any costs incurred to health and longevity. The wide lens of evolutionary theory focuses on the ultimate goal of leaving descendants, putting the costs to health and longevity into a broader perspective. In contrast, the Adverse Childhood Experiences Study suggests that individuals suffer “*disrupted neurodevelopment”* and “*social emotional and cognitive impairment”* following exposure to adverse and traumatizing environments during development, which it empirically demonstrates is associated with later costs to health and longevity (95, 96). Here, the dominant view of the medical model leads to the assumption of pathology, foreclosing an interpretation of the data from an evolutionary perspective.

#### 2.5.1. Reframing the adverse childhood experiences study

A recent study robustly challenges the commonly accepted interpretation of data from the Adverse Childhood Experience Study (118). Looking again at the ACE study but from a Life History Theory perspective, the original seven categories of adversities: physical, sexual, emotional, and domestic abuse, and parental substance use, mental illness/suicidality, or incarceration, indicate harshness and unpredictability in the early environment. Life History Theory would predict an individual who encountered four or more of these categories to likely be shepherded onto a fast life trajectory. This may manifest in accelerated development, precocious puberty, early menarche, sexual debut, and first birth, and potentially more offspring with mixed paternity (101, 112, 118). Empirical data testing hypotheses generated from Life History Theory indicate that environmental unpredictability and harshness in adolescence are associated with faster life history strategies (111) and early first pregnancy (119). Likewise, low life expectancy associates with earlier reproduction (120); early experiences of threat are associated with early puberty (121) and low birth weights, younger age at first birth and shorter duration of breastfeeding (an indicator of parental investment) occur more frequently in deprived neighborhoods^1^ compared with affluent ones (114). Empirical data from the Adverse Childhood Experiences Study and associated studies similarly show that encountering more than four adverse childhood experience categories—indicating a harsh and unpredictable environment—increases the likelihood of early sexual contact (122), teen pregnancy and fetal death (123), unintended first pregnancy (124) acquiring a sexually transmitted disease (125), having more than 50 sexual partners (96), developing heart disease, lung disease, cancer, and stroke (96), and dying early (97).

The data reveal that individuals exposed to harsh and unpredictable environments during development engage in earlier, riskier, and higher levels of reproductive activity which associates with poor health outcomes and shortened lifespans. The data support a life history model, in which under harsh unpredictable conditions, reproduction will be prioritized at the expense of health and lifespan. The data do not provide evidence for the claims of “*disrupted neurodevelopment”* or “*social emotional and cognitive impairment”* that are assumed to mediate the journey from adverse experiences in childhood to disease and early death as shown on the well-known ACE pyramid (95, 96). The fast life strategists' harsh, unpredictable environment generates fleeting, unpredictable opportunities and threats, disposing them toward a present-time orientation, which exploits immediate and short-term gains and disregards undesirable future consequences that, due to the risk in the current environment, may never eventuate (115). Taking opportunities to engage in, for example, earlier, riskier, and higher levels of sex with multiple partners that may lead to unintended pregnancies is usually stigmatized as “promiscuous behavior”, yet this fast life strategy is rational from an evolutionary perspective (109). The Adverse Childhood Experiences study interprets the adaptations of the fast life strategist as disruptions, dysfunctions, and impairments. Hertler opposes this interpretation: “*Through any application of life history evolution, the social, emotional, cognitive, and health effects associated with adverse childhood experiences transition from putative markers of dysfunction to integrated adaptations to ecologies”* (p. 3) (118). The Adverse Childhood Experience study pathologizes the fast life strategist. From an emerging perspective of Life History Theory, Hertler reasonably asks: “*Should medical and clinical practitioners, without reference to evolutionary trajectories and ecological context, reflexively pathologize patients and parents with elevated ACEs”* (p. 10) (118)?

#### 2.5.2. Neglecting evolutionary perspectives leads to default assumptions of pathology

In the absence of evolutionary adaptationist perspectives, the default pathologization of outcomes judged as undesirable is widespread. For example, the publication of an influential review in 1999 (the year following the publication of the ACE study) highlighted that chronic restraint stress led to dendritic atrophy of hippocampal neurons in rats, suppressed neurogenesis, and contributed to neuronal death, overwhelming adaptive plasticity leading to “*permanent damage*” in the hippocampus (p. 118) (126). Subsequently, the American Academy of Pediatrics published a technical report for clinicians introducing them to the term “*toxic stress”* which is linked to adverse childhood experiences, and encouraged them to view adult diseases “*as developmental disorders that begin early in life”* (p. 232) (127). Uncritical acceptance of “toxic stress” as the biological link between early adversity and poor health and social outcomes followed. A search for “toxic stress” for the 20 years prior to this publication (1991–2011) returns seven million results, and for the 20 years post-publication (2012-present) returns 200 million results. Toxic stress narratives are widely published (128–132), linked to some trauma-informed approaches (133, 134), and taught in many trauma-informed training programs including at the Center on the Developing Child (135) and in schools in the UK (136). An important addendum to this explication of “toxic stress” is to draw attention to the outcome of a study that replicated the original chronic restraint stress protocol that led to dendritic atrophy of hippocampal neurons. This study also looked at the impact on neurons in the amygdala. The authors noted that “*chronic stress induces contrasting patterns of dendritic remodeling in hippocampal and amygdaloid neurons*” (p. 6815) (137). This study suggests a nuanced and adaptive response to glucocorticoid-mediated stress, rather than stress presenting a broad “toxic” challenge to neurons, as had been originally assumed. One possible adaptive explanation is that stress may induce a functional and adaptive transition in the location of information processing similar to other stress-induced changes (138, 139).

Inattention to an evolutionary perspective also results in the unwitting pathologization of the fast life strategist through diagnostic bias, which only becomes visible from a life history perspective (140). Fast life strategists are often diagnosed with personality disorders (141, 142). A pre-requisite for an appreciation of Hertler's critique of diagnostic bias is an appreciation of how the study of life history variation in humans has developed over the last 40 years. In 1985, Rushton asserted “*The more* [of a slow life strategist] *a person is, the more likely he or she is to come from a smaller sized family, with a greater spacing of births …and more intensive parental care. Moreover, he or she will tend to be intelligent, altruistic, law-abiding, behaviourally restrained, maturationally delayed, lower in sex drive, and longer lived* (p. 441) (143). Although open to conceptual challenges (144), life history variation in humans has revealed that life history shapes personality, qualities, social and cultural values, our habitual focus on short- or long-term outcomes and, accordingly, our behaviors (106, 107, 111). Just as evolution does not shape us for health and happiness neither does it preferentially shape our personalities, traits, habits, values, and behaviors toward what is culturally desirable and prosocial, unless, in our ecological niche, those traits serve survival and reproduction. Accordingly, prosocial and culturally desirable traits: social compliance, stable attachments, self-control, agreeableness conscientiousness “… *altruism and affiliation, risk aversion and inhibition, as well as future oriented thought and delay of gratification”* (p. 2) (140) are traits associated with slow life strategists (115). In addition, social values, of cooperation and empathy, cultural values, of home ownership, having a savings account, and using contraception are associated with slow life history strategies (106, 111). Contemporary studies reveal that (pro-social) compliance with COVID-19 precautions is popular among slow life strategists (145, 146).

## 3. A non-pathologizing alternative: introducing the Neuroplastic Narrative

The medical model is premised on pathology, but psychiatry has extended the assumption of pathology beyond its verifiable scope. As yet there are no valid, reliable biomarkers for diagnosing mental illness (147). Considering the increasing evidence for ecological adaption, the future identification of clear biomarkers of disease is not likely. Wakefield notes, “*Variations in brain structure and functions are omnipresent, and which variations are relevant to pathology is not obvious from the nature of the brain structures themselves”* (p. 127) (148). However, sensitive to the suffering felt by their patients, some psychiatrists articulate a commitment to “*defending the reality of psychiatric illness”* (p. 6) (149). This sympathetic view perhaps conflates suffering and pathology, however, suffering does not necessarily imply the existence of a pathological state (5, 103).

### 3.1. The Neuroplastic Narrative complements Life History Theory

Life history theory illustrates that suffering can be, and often is, a by-product of a functioning adaptive strategy, not a pathology, disorder, or malfunction. It is crucial to understand that both the fast and slow strategies are successful in evolutionary terms. One is not more successful than the other, and neither strategy is pathological *per se*, although a fast life strategy is less desirable and is attended by suffering because it emerges from living in a harsh and unpredictable environment that is insecure and emotionally, psychologically, and physiologically dysregulating. Consequently, a fast life strategist is motivated to seek swift relief from emotional and physiological dysregulation and immediate gratification, driving short-term decisions that often compound their medium- and long-term stress, distress, suffering, and eroding their health and wellbeing. A typical history of a fast life strategist contains trauma and multiple Adverse Childhood Experiences that were recognized in the original ACE study to drive harmful “*behaviours such as smoking, alcohol or drug abuse, overeating, or sexual behaviours that may be consciously or unconsciously used because they have immediate pharmacological or psychological benefit as coping devices”* (p. 253) (96). Slow life history strategists are fortunate because they do not suffer the antecedent ecological conditions of a fast life history (complex trauma and adversity) that drive fast life short-term decisions that are so costly to health. Fast life history strategists suffer and make decisions that compound and exacerbate their suffering for which they are harshly judged and/or pathologized. “*Nobody likes suffering, but suffering may just have been the cost of reproduction in risky or uncertain environments. When the future was risky, the most evolutionary adaptive pattern was to be hyper-vigilant, to grab hold of whatever resources were available and to convert them into children as quickly as possible—to live life as if there is no tomorrow. The costs in terms of physical health and emotional well-being are often so desperate that we call these pathways “pathological”, but from an evolutionary perspective, had our ancestors not paid these costs, we would not exist.”* (p. 179) (117). By invisibly ushering us through development and towards the most successful life history strategy for survival and reproduction in our niche, our neuroplastic mechanisms safeguard our genetic line whether we develop in harsh and unpredictable or benevolent and predictable environments despite shortening life expectancy and perpetuating suffering for people who grew up in adverse and traumatizing environments. By embedding our experience in our biology and calibrating us to our environments, our neuroplastic mechanisms are functioning as they are evolved to do. They do not make a distinction between experiences in privileged (benevolent and predictable) and traumatizing (harsh and unpredictable) environments, both are embedded, calibrating us to and for more of the same kinds of experiences, shaping our anticipations. There is no pathology in the mechanisms themselves. The Neuroplastic Narrative rationally de-pathologizes the suffering of the fast life strategist and makes sense of the “real” suffering of psychiatric patients (and others) with histories of trauma and adversity by recognizing the profound, enduring consequences of biologically adapting to trauma and adversity in childhood.

### 3.2. The Neuroplastic Narrative

The Neuroplastic Narrative is an intuitive, non-pathologizing, biological explanation for the enduring emotional and psychological suffering of those of us who were affected by complex trauma or other Adverse Childhood Experiences. For better or worse, it converts the lived experience of what has happened to us into preparedness; an anticipation that what has happened may happen to us again. The Neuroplastic Narrative privileges experience and recognizes that we adapt to the threats and opportunities we have had, to better anticipate the threats and opportunities we are likely to have. Neuroplastic mechanisms act proximately to biologically embed experiences gained during development within an ecological niche, which gradually specify a life history strategy that ultimately calibrates the individual for survival and reproduction within the same or a similar ecological niche (see Figure 1). The Neuroplastic Narrative recognizes that experiences shape brains; a powerful and pivotal insight that can be drawn to both explain the impact of experiences from the past that lead to suffering and to identify what experiences may be needed now and in the future that will alleviate that suffering.

The Neuroplastic Narrative is articulated as an alternative framework to the Medical Model, substituting the pathological for the ecological. The narrative is aimed at several audiences: interested people with histories of trauma and adversity seeking meaning to their suffering beyond it being symptomatic of a mental illness; the professionals who encounter them; researchers for whom it may offer a novel perspective; and for the wider community. Simply stated, the Neuroplastic Narrative asserts that experiences gained in the ecological niche of our development are embedded by neuroplastic mechanisms shaping brains and calibrating physiologies for survival and reproduction within that (or a similar) ecological niche. Mechanisms of plasticity have evolved over evolutionary time which allows individuals to adapt to their ecological niche during developmental time. The infant arrives in the world with approximately 86 billion neurons (150) making connections at a rate of 40,000 synapses a second during early post-natal life when every experience is novel until it is repeated (151). The infant's experiences—for better or worse—are biologically embedded by evolved neuroplastic mechanisms, epigenetics, neurogenesis, and synaptic and white matter plasticity. What emerges as a result of these adaptive processes is a brain and physiology that is simultaneously a record and an anticipation of the experiences that have most often been repeated. The process of adaptation prepares us for experiences, relationships, and life in a future ecological niche that closely resembles the experiences, relationships, and life in our past ecological niche. We do not so much remember our pasts, as neurobiologically and physiologically anticipate their recurrence. The impact of past adverse and traumatizing experiences, relationships, and ecologies coupled with our biologically embedded but not necessarily conscious anticipation of similar ecologies and experiences in the future engenders suffering that is real, valid, and can be effectively responded to, but is not pathology *per se*. The Neuroplastic Narrative describes the normal processes and mechanisms of adaptation to the ecology of development, recognizing that as ecologies of development are diverse, ranging from the severely abusive or profoundly neglectful and potentially fatal to those that are facilitative and benevolent, there is extreme diversity in the experiences that are embedded. The diversity of ecologies of development, therefore, creates brains and physiologies which are commensurately “*neuroecologically diverse”* adapted for the environment by which they have been specified (Peckham and Hamilton under review). The narrative of neurodiversity is intended to appreciate rather than pathologize differences (152, 153). The term neuroecological diversity, which reflects neural and physiological differences resulting from different experiences is also intended to refocus judgment away from what may be wrong or lacking in the individual and direct it toward their ecological context, thus drawing attention to inequities and the need for social justice (Peckham and Hamilton under review).

Life History Theory ultimately explains that fast life traits consistently lead to poor health outcomes and shortened lifespans because the fast life strategy of trading resources away from health toward reproductive effort is evolutionarily successful in harsh, unpredictable environments. The Neuroplastic Narrative articulated here for the first time offers a complementary proximate explanation: how experiences gained in the developmental niche lead to physiological and neural changes and the formation of traits, that enact the life strategy likely to be most successful in the ecological niche encountered. The complementary relationship between Life History Theory and the Neuroplastic Narrative is supplemented and extended by the incorporation of Attachment Theory.

### 3.3. Situating the Neuroplastic Narrative in relation to Attachment Theory

The relational bond of attachment between an infant and their mother (or caregiver) is most readily observed while it is forming in infancy and toddlerhood when the infant is wholly reliant on the closeness and care of their mother for survival. Less readily observed are the profound and enduring effects this early adaptation to their mother has on the individual's capacity to regulate themselves and to relate to others throughout their life. The formation of a relationship is fundamental for the infant's survival, and the critical adaptations the infant makes to attach to their mother so that they may survive or can thrive in infancy profoundly shapes the relational context in which reproduction and child-rearing occur at maturity.

In 1958, Bowlby published *The Nature of the Child's Tie to his Mother*, an ethological theory that postulated five instinctual infant behaviors (sucking, clinging, following, crying, and smiling) that when integrated and directed to a mother figure became manifestations of “*attachment behavior”* (154). He suggested the infant's attachment behavior, the tie to their mother, had survival value because the instinctual attachment behaviors elicited reciprocal responses from the mother that brought her close and elicited her care. “*I wish to emphasize that it is a main part of my thesis that each of the five instinctual responses which I am suggesting underlie the child's tie to his mother is present because of its survival value. Unless there are powerful in-built responses which ensure that the infant evokes maternal care and remains in close proximity to his mother throughout the years of childhood he will die — so runs the thesis. Hence in the course of our evolution the process of natural selection has resulted in crying and smiling, sucking, clinging and following becoming responses species-specific to Man”* (p. 370) (154). Bowlby orientates the reader to the generic aspects of attachment, behaviors that have proved survival positive and have thus been retained by natural selection over many lifetimes. Further adaptation specific to the caregiver occurs within developmental time. Wells notes that developmental plasticity is maximand during the period that the infant is physiologically reliant on their mother which is coincident with her being the mediator of and buffer for, all ecological stressors and influences, thus any adaptation that occurs that will be to her (155). Developmental plasticity occurs when “a *proximate mechanism tracks the state of the environment and calibrates the phenotype accordingly”* (p. 44) (156). So, as the mother is the putative target of the infant's adaptations, proximate mechanisms in the infant track her responses to their attachment behaviors, and those that promote proximity and elicit her care are noted and favored. Over many repetitions, this becomes an habitual pattern of attachment.

Attachment Theory presented an opportunity for Life History theorists to address the perplexing problem of how infants could calibrate their life history strategy to extrinsic morbidity–mortality rates in the wider environment given that “*…children cannot directly perceive local mortality rates”* (p. 7) (102). Belsky *et al*. proposed that “*rearing context shapes life history”*, suggesting that the early caregiving environment was sufficiently predictive of the environment at maturity that early relational experiences did the work of shepherding development toward the appropriate adaptive reproductive (life history) strategy (p. 649) (101). Chisholm similarly proposed that the quality of parental care and nurture indexed the local mortality rates in the environment observing that “*…universal sources of parental stress are the routine social and environmental causes and correlates of high mortality rates, poverty, exploitation, hunger, disease, and war and their accompanying fear and hopelessness”* (p. 7) (102). In this way, parents would convey information about the environment to their infant through the quality of their caregiving enabling the infant to calibrate their life history strategy accordingly. Life History Theory positions Attachment Theory as a proximate mechanism, proposing that the quality of attachment—or lack of attachment—indicates how harsh and unpredictable the wider environment is, so the life strategy appropriate to the environment can be specified. Here, I propose similarly to above, the biological embedding of experience is the interface between the Neuroplastic Narrative and Attachment Theory. Neuroplastic mechanisms act proximately to biologically embed relational experiences gained during development with a caregiver, which gradually specify an attachment pattern. The Neuroplastic Narrative as the transducer of lived experiences including relational experiences into meaningful biological change is positioned as a more fundamental proximate explanation complementary to and nested within Attachment theory. Attachment Theory is itself a more fundamental proximate explanation complementary to and nested within Life History Theory (see Figure 1). To elaborate on the complementary relationship between these interdependent evolutionary theories what follows is a brief explanation of how attachment and a life history strategy may be specified in individuals positioned at the extremes on the continuum of ecological niches. Consider the infant emerging into the world, “*thanks to the human nature he inherits, the infant is predisposed to be interested, amongst other things, in the feel at his lips of something warm, moist, and nipple-like, or the sight of a pair of sparkling eyes, and is so made that he responds to them in certain characteristic ways, to the one by sucking and to the other by smiling”* (p. 362) (154). How these “instinctual behaviors” are responded to by the postnatal environment profoundly shapes how the rest of the infant's life is lived.

#### 3.3.1. Becoming a slow adapted, securely attached slow life strategist

Emerging into a benevolent and predictable environment, the infant's instinctual behaviors are reliably met with attuned, sensitive, well-resourced, and supportive parents. Crying is interpreted as communication of an unmet need and the parents cycle through possible causes, attending cyclically to hunger, discomfort, tiredness, pain, and fear. The parents begin to discern hungry cries from tired cries, honing and improving their responses to the expressed need. A routine gradually comes into focus. The impact of loud bangs, specters, losses, and all things upsetting are soothed by one or other parent. The emergence of smiles and prosodic proto-conversations and other playful reciprocities elicit mutual delight (157). The infant experiences agency, learning that crying brings a soothing someone; pointing makes them look somewhere; refusing what's not wanted evokes the efforts of another to identify what is. The infant's staple experiences are of benevolent care in a predictable world where their emotions are regulated and they, the infant, matter and matter enough that they can bring who is needed into proximity and elicit their attention and care. Neuroplastic mechanisms embed these formative and repeated ecological, relational, and personal experiences of predictability, relational safety, and agency into the infant's and then the child's biology. Repeated experiences incrementally and simultaneously specify and generate psychobiological anticipations, *internal working models* of the self and the world. These are *internal working models* in both the attachment sense (158) and as defined by complex adaptive systems theory (see Section 2.4.1) (110), which for this infant, lead to a secure attachment and slow life strategy. In a predictable world, the agent who learns to plan and control it may make significant compound gains by orienting to a future when they will reap their deferred rewards. The timing of reproduction even birth itself can be planned for when the parents decide they have accrued sufficient resources to enable their parenting efforts to produce competitive offspring (see Figure 1).

#### 3.3.2. Becoming a fast-adapted, disorganized fast life strategist

**Figure 2:**
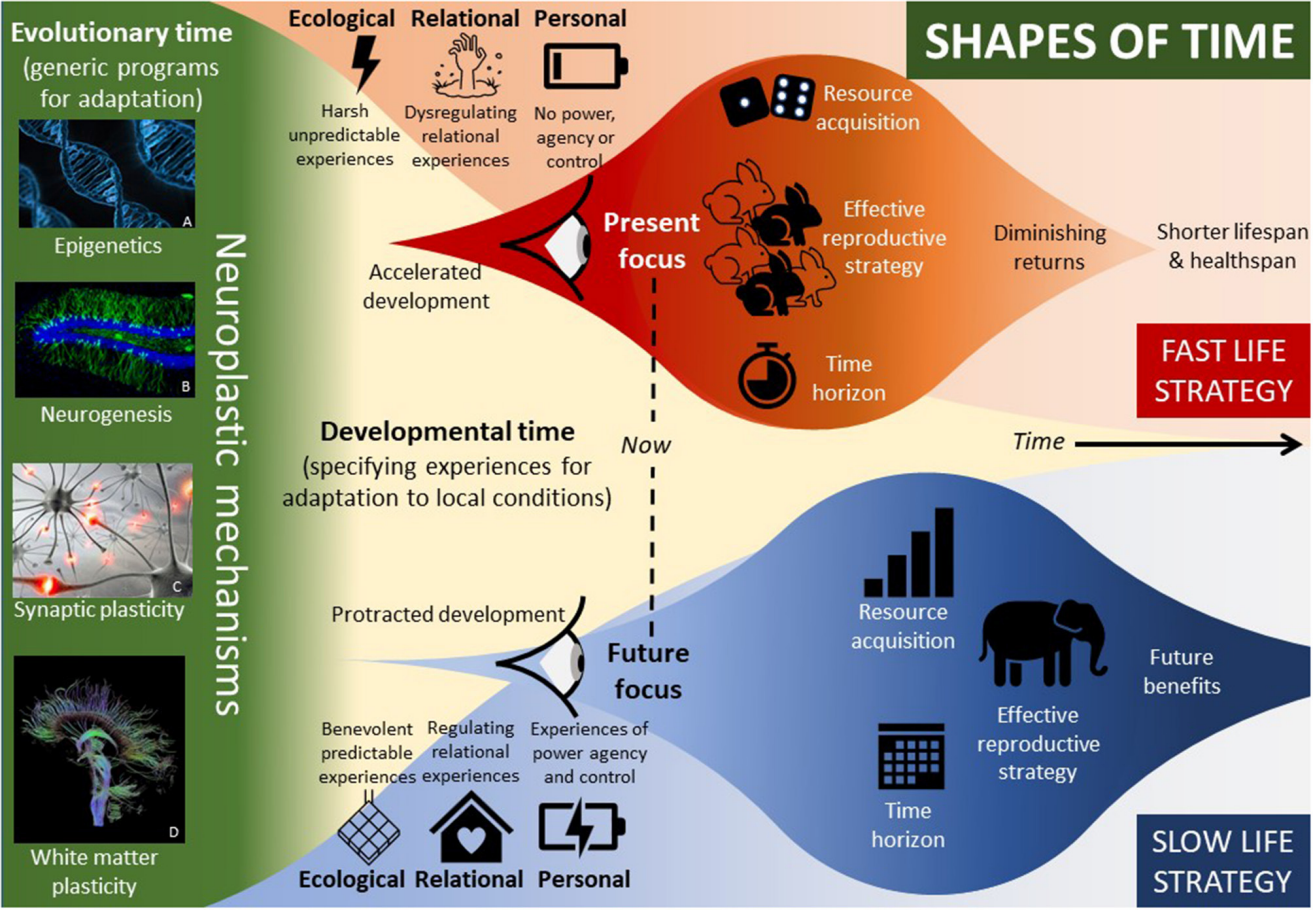
Shapes of time. Adaptation occurs over evolutionary time as generic (genetic) programs are honed by natural selection, and within developmental time as these generic programs are specified by experiences in the local environment. Evolved neuroplastic mechanisms **(A–D)** transduce experiences into biology, specifying adaptations to the local environment. Harsh and unpredictable ecologies, dysregulating relationships, and few experiences of agency and control are likely to specify a fast life history commencing with accelerated development. Benevolent and predictable ecologies, regulating relationships, and many experiences of agency and control are likely to specify a slow life history commencing with protracted development. Many aspects of the individual's traits, values, and behaviors are adapted by their experiences of the environment including their orientation to time (shown as time horizon), their strategy for resource acquisition, and the ultimate adaptation which all others serve—the strategy for reproductive success. At the extremes, fast life strategists are present-focused, opportunistic, risk takers energetically disposed toward the mating effort, and slow life strategists are future-focused, steady acquisitors energetically disposed toward parenting effort. Deferring to the future benefits the reproductive strategy of the slow life strategist in a benevolent predictable environment. Discounting the future benefits the reproductive strategy of the fast life strategist in a harsh unpredictable environment but shortens healthspan and lifespan. **(A)**: https://commons.wikimedia.org/wiki/File:Dna-163466.jpg; **(B)**: Jason Snyder Doublecortin and Hoescht Creative Commons https://www.flickr.com/photos/functionalneurogenesis/4191317925; **(C)**: Else if then. https://commons.wikimedia.org/wiki/File:R%C3%A9seau_de_neurones.jpg; **(D)**: Thomas Schulz https://en.wikipedia.org/wiki/Tractography#/media/File:DTI-sagittal-fibers.jpg.

Alternatively, an infant's instinctual behaviors may be met chaotically by parent or parents under-resourced, unsupported by family, community, and society, perhaps young, perhaps in the grip of their own unresolved traumas, and barely managing their own unmet needs. The infant's crying may elicit mixed responses of attendance and care, punishment through emotional or physical violence, or no response at all. Chaotic interruptions and demands disturb attempts to establish routine. Rather than soothing the specters, loud bangs, and losses, the parents are frequently the source of them, compounding the infant's distress. The infant cycles through their instinctual behaviors in an evolved attempt to discern a strategy that will maintain the parents' proximity and evoke their care. However, they may encounter that their evolved drive to seek proximity for safety conflicts with their experience of proximity being dangerous, precipitating a circumstance of “*fright without solution”* (p. 484) (159) and disorganizing their search for attachment (160). The infant's initiatives may go unnoticed, be thwarted, or evoke unpredictable responses, pervasively foreclosing their opportunities to experience themselves as agents. Their staple experiences are of a harsh and unpredictable world of emotional dysregulation where they are unable to rely on their agency or capacity to bring a parent into proximity from whom they can evoke care. Neuroplastic mechanisms repeatedly embed these formative experiences into the infant's and child's biology, accelerating development and building an *internal working model*. The model psychobiologically anticipates the future from the lived experience of the past and thereby specifies disorganized attachment and a fast life strategy. Whereas agency and future focus are key for resource acquisition and reproductive success in the predictable life of the slow life strategist, they are nullified in the unpredictable world of the fast life strategist. In an unpredictable world, the likelihood of any particular outcome occurring is unrelated to the intentional actions or plans of a putative agent so appeals to gain rewards or avoid punishments in the future gain no traction. The most rational strategy in a harsh and unpredictable world, or one made so by the arbitrary will of a punishing or neglectful authority, is accelerated development and maximizing reproductive effort, facilitated by opportunism, a present focus, and audacity (see Figure 2).

The Neuroplastic Narrative demonstrates how experiences are embedded in biology providing proximate explanations for Life History Theory and Attachment Theory. As such, it also provides a non-pathologizing biological foundation for trauma-informed understandings.

## 4. The Neuroplastic Narrative is foundational for trauma-informed, adverse childhood experience-aware understandings

### 4.1. Core features of trauma-informed understandings

In 1979, The Drama of the Gifted Child by the Swiss psychoanalyst Alice Miller was published. It presented the central thesis that the cost of unremembered and unresolved abuse and trauma in childhood is repetition, as harm is perpetuated either onto others, emerging as crime or the repetition of abusive parenting; or the self, emerging as mental illnesses or addiction. Later in 1992, Judith Lewis Herman published Trauma and Recovery: from Domestic Abuse to Political Terror. Herman asserted that “*Chronic abuse causes serious psychological harm*” (p. 116) (19). Subsequently, Sandra Bloom published The Sanctuary Series which shared the premises of Miller's and Herman's work; that childhood trauma is a “*causative factor”* in psychiatric and social disorders and that “*post-traumatic reactions are essentially the reactions of normal people to abnormal stress”* (p. 135) (20). This shift to privileging the experiences of the individual in their ecological context and normalizing their response to it is the *sine qua non* of trauma-informed approaches. This paradigm shift, simply captured by Joseph Foderaro moves away from understanding “what's wrong with you?” toward understanding “what's happened to you?” (p. 9) (161). The growing appreciation that something has happened—that experiences of complex trauma and adversity mark the lives of most people engaged with mental health and addiction services prompted Harris and Fallot to call for services to become “*trauma-informed*” (162). They noted that complex trauma impacts identity and organizes experience foreshadowing findings from neuroscience. In his commentary on Teicher and Samson's 2016 landmark review “Enduring neurobiological effects of childhood abuse and neglect”, van der Kolk summarizes that “*following abuse and neglect, the world is experienced with a different nervous system”* (p. 267) (163). In 2014, the U.S. Department of Health and Human Services Substance Abuse and Mental Health Services Administration published SAMHSA's Concept of Trauma and Guidance for a Trauma-Informed Approach which set out a definition of trauma (3Es); assumptions about trauma for organizations (4Rs); six key principles of a trauma-informed approach and 10 domains for organizational implementation (164). These trauma-informed approaches are unified in validating the profoundly shaping and enduring impact of experiences of complex trauma in the lives of trauma survivors. Noting that shame and shame dynamics are an inherent part of trauma, Dolezal recently proposed extending trauma-informed approaches to include shame-sensitive practice (165).

### 4.2. The Neuroplastic Narrative and trauma-informed understandings of suffering

The premise of the Neuroplastic Narrative is that experiences shape brains and physiology in ways that are meaningful and adaptive and serve survival and reproduction in the environment that has been encountered. What has happened becomes a neurobiological anticipation of what may happen. The Neuroplastic Narrative thus provides a biological foundation for a trauma-informed understanding that privileges the person's lived experiences. Trauma-aware writers foreshadowed that traumatic experiences had a profound biological effect. “*Traumatic events appear to recondition the human nervous system”* (p. 36) (19) and “*the reactions to trauma are based on biological changes in the mind and body over which children have no control”* (p. 3) (161). Our understanding of precisely how experiences of trauma and adversity shape developing brains is in its infancy as research has been dominated by the search for genes and pathological mechanisms that cause putative mental illness. However, the premise, common to trauma-informed approaches and the Neuroplastic Narrative that complex trauma, neglect, socioeconomic status, and adverse experiences, shape brains and physiology is firmly established (1–3, 37, 38, 85, 166–168).

Hostile, dangerous, and unpredictable early relationships not only fail to provide the ordinary physiological and emotional regulation needed by the infant/child but are of themselves dysregulating. Compounding, repeated experiences of unrelieved emotional and physiological dysregulation create a need for proportional measures that will provide relief and regulation. Herman eloquently captures the task of a child adapting to an abusive environment: “*The pathological environment of childhood abuse forces the development of extraordinary capacities … she must find a way to develop a sense of basic trust and safety with caretakers who are untrustworthy and unsafe. She must develop a sense of self in relation to others who are helpless, uncaring, or cruel. She must develop the capacity for bodily self-regulation in an environment in which her body is at the disposal of others' needs, as well as a capacity for self-soothing in an environment without solace. She must develop the capacity for initiative in an environment which demands that she bring her will into complete conformity with that of her abuser and ultimately, she must develop a capacity for intimacy out of an environment where all intimate relationships are corrupt, and an identity out of an environment which defines her as a whore and a slave”* (p. 101) (19). These paradoxical and extreme circumstances generate commensurately extreme and paradoxical self-regulating behaviors of self-harm, sexual self-harm, restricting and purging, risk-taking, suicide attempts, and substance or process addictions; all precipitating and/or resolving a familiar state of extreme autonomic arousal through endogenous means.^2^ Extreme self-regulating behaviors arising from adapting to survive in an extreme, dysregulating environment are framed by the medical model as symptoms indicating an underlying pathology (169, 170). However, if these behaviors that regulate extreme states are evidence of self-preservation and resolve a problem (albeit by creating a different but perhaps preferable one) as trauma survivors and writers suggest, they are survival-serving and adaptive (5, 161, 170, 171).

Experiences in childhood such as those described above are shaming. Feelings of shame are evoked relationally when a connection conveying an emotional tone is sought, but not found. Thus, shame arises if we look to the other for acknowledgment, respect, warmth, approval, validation, attention, and empathy, to relate to us and find instead only absence. Mollon describes shame as “*a hole where our connection to others should be”* (p. 23) (172). Childhood trauma takes place in such holes. When the parent or adult fails to recognize or denies the subjectivity of the child, or their subjective experience, and/or treats the child as an object to meet their own subjective needs, intersubjective relating is void, and the void is shame (173). Dominance and subordination in relationships indicate shame dynamics. Shame feels unbearable, like not mattering, being fundamentally flawed, damaged or toxic, wrong to the core, and wanting to disappear (174). In a manner described by the Neuroplastic Narrative, repeated shaming experiences generate an anticipation of shame and the experiences that trigger it. This anticipation may be referred to as toxic or chronic shame where the shame experience organizes a person's identity and life around avoiding the anticipated and feared shame experience (165, 172). Various characterological defenses (175) or defensive scripts (176) may be employed to strategically manage shame transforming it into numbing withdrawal, violence toward self or others, or relentless perfectionism and achievement (177–179). Unsurprisingly, shame causes or correlates with relationship and employment difficulties, self-harm, substance and process addictions, disordered eating, and diagnoses that associate with shame manifestations (180). Shame-driven distress and suffering is common across experiences of trauma and adversity and many diagnoses of mental illness.

Herman considers post-traumatic stress disorder (PTSD) to be a shame disorder (181). She originally proposed a new diagnosis for the “*syndrome that follows upon prolonged, repeated trauma …. complex post-traumatic stress disorder”* (p. 119) (19). PTSD refers to an experience or experiences that have been traumatic and Herman, frustrated by mental health professionals presuming an “*underlying psychopathology”* rather than recognizing “*a response to an abusive situation”* proposed complex PTSD to validate the link between presenting symptoms and the traumatic experience (19). An explicit recognition of their experience as traumatic is validating for many people with trauma histories receiving a diagnosis of CPTSD echoing Bloom's words, “*post-traumatic reactions are essentially the reactions of normal people to abnormal stress”* (p. 135) (20). However, Dearing and Tangney draw attention to the possibility that “*Therapists can unintentionally shame clients by focusing on or reifying a psychiatric diagnosis. Clients may end up feeling that the therapist sees them not as a person but objectified as a diagnostic category*” (180). This may be compounded if the diagnosis is experienced or seen by others as a stigmatizing marker, compounding shame (182). Of note, a diagnosis of psychopathology may be the necessary ticket for accessing treatments, consultations, and funding, thus the person, by accepting a diagnosis carries a component that the service infrastructure needs to operate and becomes functionally objectified. The Neuroplastic Narrative offers an alternative approach that also validates and normalizes “*the reactions of normal people to abnormal stress”* but avoids the shaming potential of diagnosis (p. 135) (20).

The Neuroplastic Narrative recognizes the evolved rationale of experiences shaping brains and calibrating physiology. The “desperate times” of repeated, unpredictable, profoundly dysregulating, and shaming early traumatic experiences set the neurophysiological stage for a fast life strategy that calls for “desperate measures”. These include behaviors or substances (exogenously taken or endogenously produced) that regulate these extreme states, providing much-needed relief. Even when the health-compromising long-term costs and consequences of the “desperate measures” are rationally understood by the fast life strategist, pain, including emotional pain and immediate need, compel an orientation to the self, in the present where any appeal to what is rational in the long-term cannot gain traction (183). The difference between the fast life strategist and the slow life strategist is one of fair fortune. The slow life strategists do not need to develop “desperate measures” having not experienced the “desperate times” that demand them. The difference between fast and slow adapted brains and physiologies indicates *neuroecological diversity*, differences that arise as a result of neuroplastic mechanisms biologically embedding different experiences, not brains and physiologies that are normal (slow adapted) and pathological (fast adapted) (Peckham and Hamilton under review).

### 4.3. The Neuroplastic Narrative and trauma-informed approaches to healing

Stable and predictable relationships that counter the survivor's anticipation of relational harms heal complex trauma (161). These must be relationships where attention is paid to safety, trustworthiness, empowerment, choice, and collaboration (184) and an intentional awareness must be brought to ensure that the traumatizing features of relationships are not repeated, further re-traumatizing survivors (161). “*Recovery can take place only within the context of relationships; It cannot occur in isolation. In her renewed connections with other people, the survivor recreates the psychological faculties that were damaged or deformed by the traumatic experience. These faculties include the basic capacities for trust, autonomy, initiative, competence, identity, and intimacy. Just as these capabilities are originally formed in relationships with other people they must be reformed in such relationships*” (p. 133) (19). The critical context of relationship is underscored by relational psychotherapist Pat DeYoung who advises that the shamed person, instead of having more dysregulating, disintegrating shaming experiences needs repeated validating and responsive experiences with a regulating empathic other, allowing the shamed self to be integrated (174). These healing relational experiences alleviate the shame and other affect-based distress and suffering in the moments as they occur, and the compound effect of repeated healing relational experiences dynamically updates the trauma survivors' distressing and negative anticipations (inner working model) of relationships (185).

The fundamental premise of the Neuroplastic Narrative is that experiences shape brains. It follows that “new experiences” can be used to heal or mitigate the impact of previous experiences; psychotherapy being an obvious candidate for “new experience”. The brain-changing nature of therapeutic interaction was foreshadowed by Nobel Prize winner Eric Kandel who hoped that neuroscience would reinvigorate psychoanalytic research. “*Insofar as our words produce changes in our patient's mind, it is likely that these psychotherapeutic interventions produce changes in the patient's brain. From this perspective, the biological and sociopsychological approaches are joined”* (p. 466) (186, 187). Imaging studies reveal the prescience of his words. Story-telling, the act of meaningfully engaging with a spoken narrative, couples the brains of speakers and listeners together, and the stronger correlation between the brain activity of the speaker and listener, the better the communication between them (188). De Young writes “*the ‘different experience' that generates emergent change is* ***heightened affective relational experience***” (p. 150) (174) (original author's emphasis). Schore develops this narrative “*In “heightened affective moments” of a psychotherapy session the intuitive therapist implicitly “surrenders”* …to receive her patient's right brain emotional communications; interbrain synchronization of right temporoparietal regions occurs, which “… *allows the empathic therapist to emotionally* ***recognize*** *the patient and enables the patient's right brain subjective self to emotionally experience* ***feeling felt*** *by the therapist”* (p. 14) (189). Anna O's “talking cure” is no less of a biological intervention than any pharmacological agent; the patience and devotion to the task of listening effects the treatment (190). “*There is no longer any doubt that psychotherapy can result in detectable changes in the brain”* (p. 155) (191).

As the Neuroplastic Narrative is a biological theory of how experience shapes our brains, there is a role for medication that is not conceptualized as a cure or treatment for a diagnosed mental illness, but for their much less recognized, effective, and broad function as modulators of neuroplasticity. Psychoactive medications, antipsychotics, and antidepressants alter our epigenome (192, 193) and neurotransmission, either synaptic or white matter plasticity (194), and some may influence molecules that act broadly, such as brain-derived neurotrophic factor (BDNF). BDNF is a molecular hub of neuroplasticity; its gene transcription is regulated by early life experience (195) and BDNF itself facilitates synaptic and white matter plasticity, through highly complex dynamically regulated networks with other signaling molecules such as those involved in mediating the stress response (75, 196, 197). Accordingly, as BDNF is a molecular transducer of experience into plastic changes in the brain, it is implicated in numerous psychiatric disorders (198) and mediates the effects of treatments from ketamine to exercise, including antipsychotics (199), antidepressants (200–203), and MDMA (204). The authors speculate that MDMA along with other antidepressants may reopen a window of neuroplasticity (204) allowing the experiential aspect of “manualized therapy” to take effect.

Pharmacologically opening a window of neuroplasticity effectively allows experience in through that window, which offers both the possibility of having a new experience that contradicts previous experiences or repeating and confirming already known experiences. Opening a window of neuroplasticity is a “double-edged sword” as the experience, made more potent by pharmacological means, can be either supportive or adverse, potentially making matters worse (33, 205). For this reason, prescribers of these medications should attend to the possibility that people seeking pharmacological support for low mood may be experiencing ongoing adversity or trauma, for example, in domestic or workplace settings. If this is occurring, then these experiences in combination with pharmacological enhancers of neuroplasticity would consolidate rather than mitigate the impacts of adversity and trauma. Grawe, author of the ground-breaking book Neuropsychotherapy writes, “*From a neuroscientific perspective, psychopharmacological therapy that is not coordinated with a simultaneous, targeted alteration of the persons experiences cannot be justified”* (p. 5) (34).

### 4.4. Implications for formulation and research

Neuroscientific evidence detailing how experiences shape brains is accruing and gathering momentum. These studies begin from a premise of curiosity about how particular experiences, such as threat, neglect, abuse, or attachment style, or constellations of experience, such as low socioeconomic status, shape the structure and function of the brain, physiology, and immune system. These research approaches are more fundamental than the medical model that takes pathology as its premise thereby adding in an extra layer of explanation and requiring “mental illnesses” to be biologically defined—a Holy Grail in psychiatry that has remained elusive for decades. To quote two former directors of the U.S. National Institute of Mental Health, we should abandon the “*fool's errand”* of seeking out the neurobiology of our diagnostic criteria and “*rethink this whole approach”* (p. 3) (206), perhaps by returning to fundamentals. Hughling Jackson's second law was “*the study of the causes of things must be preceded by the study of the things caused”* (p. 64) (207). The Neuroplastic Narrative, by privileging experience and recognizing its biological potency, obviates the need for the premise of pathology while promoting basic descriptive biological research and “study of the things caused”.

Additionally, for survivors of complex trauma or adversity, the Neuroplastic Narrative brings a long-overdue reconciliation with a biological approach. The Neuroplastic Narrative validates the profound and enduring biological impact of experiences providing an alternative narrative to pathology and diagnosis. This shift in perspective generates new research possibilities including the possibility for radical and potentially fruitful co-production in the biological sciences between survivors of complex trauma and scientists who, alongside the renowned neuroscientist Joseph LeDoux, recognize that there is validity and value in drawing on people's self-report of subjective experience to inform and guide research in the neurosciences (208).

The Neuroplastic Narrative, in addition to the Medical Model, provides a more collaborative scope for formulation with people seeking to understand their distress and suffering because it offers choice, a cornerstone of a trauma-informed approach. The assumption of pathology and a diagnosis is disempowering and oppressive for some people with complex trauma histories. Russo writes about “*the silence imposed on us by psychiatric discourse”* (p. 59) and Filson notes that “*in the context of the medical model the story we learn to say is that we are ill”* (p. 22) (18). In the absence of a known truth about the fundamental cause of their difficulties, a framework that makes meaningful sense of them is helpful. For some people, this may be the medical model, and for others, the Neuroplastic Narrative, but their choosing empowers them.

## 5. Conclusion

The recent WHO “*Guidance on community mental health services”*, reports that services demonstrate “*an entrenched overreliance on the biomedical model in which the predominant focus of care is on diagnosis, medication, and symptom reduction while the full range of social determinants that impact people's mental health are overlooked,”* (p. XVII) (209). The Neuroplastic Narrative, articulated here for the first time, demonstrates that the social determinants of health and mental health are biologically embedded through evolved mechanisms of neuroplasticity that ultimately serve evolution's end of survival in the service of reproduction. The premise of the Neuroplastic Narrative is that experiences shape brains and physiology in ways that are meaningful and adaptive (although not yet fully characterized), and which evolved to serve our survival and reproduction in the early environment we encounter. The mechanisms that transduce lived experiences into these biological changes are referred to here, collectively, as neuroplastic mechanisms. Early experiences act as a life sample and are biologically embedded by neuroplastic mechanisms which build a brain and calibrate a physiological system for that environment. By so doing, experiences shepherd us onto a trajectory, an attachment style, and a life history strategy that serves our evolutionary fitness (survival and reproduction) within the niche from which the life sample was taken. As discussed, suffering that arises from ecological adaptation is not of itself pathological but is better understood as the evolutionary cost and consequence of surviving in, and adapting to, adverse experiences or environments including experiences of complex trauma. This suffering is valid and warrants our care but is not a pathology needing a cure.

The Neuroplastic Narrative is a neuroecological theory that recognizes that experiences shape brains and leverages this insight to indicate the importance of treating the suffering of the individual as well as the ecological conditions that give rise to it. Trauma-informed approaches guide practitioners and services to be alert to the current and historical context of the person seeking help, to do no further harm, and to offer restorative experiences of safety, trustworthiness, collaboration, empowerment, and choice. However, evolutionary theorists suggest that the scope of intervention should be widened to include social justice. Rather than attempting to uncouple the undesirable adaptations sculpted by natural selection from the ecological context that gives rise to them in individuals, evolutionary theorists urge us to address the antecedent conditions and inequality in our societies (109, 118). In Belsky's words, “*if we do not want this evolved developmental wisdom to manifest itself—in accelerated development and poor physical and mental health as a result of particular early-life adversities*—*then we need to change the contextual conditions that give rise to it”* (p. 244) (112). This shift from focusing on the individual to including their ecological context also means that is no longer acceptable or sufficient to treat the suffering of the individual and not respond to the traumatic antecedents of that suffering. The Neuroplastic Narrative and evolutionary understandings are in accord with clinicians and researchers calling for public health prevention of and response to complex trauma (210–212).

People who use services, clinicians, and people with histories of adversity and trauma may value the intuitive appeal of the Neuroplastic Narrative and the formal recognition of what we know to be true—that our experiences shape who and what we are, and become, in the world. The Neuroplastic Narrative's premise that experiences shape brains, offers compassion retrospectively, for past experiences that have happened and cannot be changed; and hope, prospectively, for new experiences to become embedded mitigating the impact of the earlier harmful experiences, allowing them to be modified, remembered, and therefore anticipated differently. By reclaiming biology but obviating the need to invoke pathology and therefore diagnosis, the Neuroplastic Narrative achieves a non-pathologizing, evolutionarily coherent, biological foundation for trauma-informed, ACE-aware approaches in mental health; an alternative to the medical model, and a framework for anyone who feels their struggle is not due to their being weak, or sick, but are hurt and in need of validation, understanding, and help.

## Data availability statement

The original contributions presented in the study are included in the article/supplementary material, further inquiries can be directed to the corresponding author.

## Author contributions

The author confirms being the sole contributor of this work and has approved it for publication.

## Footnotes

^1^It should be noted that deprived neighborhoods have low socioeconomic status which correlates with higher levels of almost all forms of morbidity and mortality (105).

^2^With the exception of regulation achieved through taking exogenous psychoactive substances.
